# Arsenic Contamination in Food-chain: Transfer of Arsenic into Food Materials through Groundwater Irrigation

**Published:** 2006-09

**Authors:** S.M. Imamul Huq, J.C. Joardar, S. Parvin, Ray Correll, Ravi Naidu

**Affiliations:** ^1^ Department of Soil, Water and Environment, University of Dhaka, Ramna, Dhaka 1000, Bangladesh; ^2^ Mathematics and Information Sciences, Commonwealth Scientific and Industrial Research Organisation, Waite Campus, Urrbrae, SA 5064, Australia; ^3^ Centre for Environmental Risk Assessment and Remediation, University of South Australia, Mawson Lakes Campus, Mawson Lakes, SA 5095, Australia

**Keywords:** Arsenic, Arsenic contamination, Food, Plants, *Colocassia antiquorum*, Bioavailability, Bangladesh

## Abstract

Arsenic contamination in groundwater in Bangladesh has become an additional concern *vis-à-vis* its use for irrigation purposes. Even if arsenic-safe drinking-water is assured, the question of irrigating soils with arsenic-laden groundwater will continue for years to come. Immediate attention should be given to assess the possibility of accumulating arsenic in soils through irrigation-water and its subsequent entry into the food-chain through various food crops and fodders. With this possibility in mind, arsenic content of 2,500 water, soil and vegetable samples from arsenic-affected and arsenic-unaffected areas were analyzed during 1999–2004. Other sources of foods and fodders were also analyzed. Irrigating a rice field with groundwater containing 0.55 mg/L of arsenic with a water requirement of 1,000 mm results in an estimated addition of 5.5 kg of arsenic per ha per annum. Concentration of arsenic as high as 80 mg per kg of soil was found in an area receiving arsenic-contaminated irrigation. A comparison of results from affected and unaffected areas revealed that some commonly-grown vegetables, which would usually be suitable as good sources of nourishment, accumulate substantially-elevated amounts of arsenic. For example, more than 150 mg/kg of arsenic has been found to be accumulated in arum (*kochu*) vegetable. Implications of arsenic ingested in vegetables and other food materials are discussed in the paper.

## INTRODUCTION

It is widely accepted that ingestion of arsenic-contaminated groundwater is the major cause of arsenic poisoning in arsenic-affected areas of the world, including West Bengal in India and Bangladesh. Contamination of groundwater by arsenic in the Deltaic region, particularly in the Gangetic alluvium of Bangladesh and part of West Bengal, has become one of the world's most important natural calamities. The Department of Public Health Engineering first identified arsenic in well-water in Bangladesh in 1993 (1). According to Karim (2), groundwater in the majority of wells in 60 of the 64 districts, covering approximately 118,000 sq km (nearly 80% of the country), has concentrations of arsenic exceeding the World Health Organization's limit of 10 μg/L (3), and only 30% of groundwater contains arsenic at levels below 50 μg/L, the Bangladesh drinking-water standard. Concentrations of arsenic exceeding 1,000 μg/L in shallow tubewells were reported from 17 districts in Bangladesh (4). High levels of arsenic in groundwater occur in the districts of Chandpur, Comilla, Noakhali, Munshiganj, Brahmanbaria, Faridpur, Madaripur, Gopalganj, Shariatpur, and Satkhira. High levels of arsenic have also been found in isolated ‘hot-spots’ in the southwestern, northwestern, northeastern, and north-central regions of the country (5). The reported number of people exposed to arsenic-contaminated drinking-water, exceeding 50 μg/L, varies significantly. Literature shows that the figures range from 29 million (6) to about 40 million people (7). About 7,500 patients with arsenicosis have been identified in 37 districts (8).

Efforts are being directed towards ensuring safe drinking-water either through mitigation techniques or through finding alternative sources of water. Even if supply of an arsenic-free drinking-water is ensured, arsenic-contaminated groundwater will continue to be used for irrigation purposes, posing a significant risk of this toxic element accumulating in the soil and, consequently, entering into the food-chain through plant uptake and consumption by animals and humans. Thirty to forty percent net cultivable land is under irrigation, and more than 60% of this irrigation is met from groundwater (9); thus, the risk of arsenic-contaminated water being used is high.

During the past 10 years, researchers have mainly focused on ingestion of arsenic through contaminated drinking-water, but the incidence of arsenicosis in the population is not consistent with the concentration of arsenic in drinking-water obtained from groundwater. This inconsistency has raised questions on potential pathways of ingestion of arsenic (10). According to Ahmed (3), while there is a very weak relationship between the number of patients and the average arsenic content in drinking-water at a local level, there is a stronger relationship at the regional level. These findings are consistent with observations of many researchers that people using water from the same source are not equally affected and that people from the same household ingesting water from a common tubewell may not be equally affected (11).

The observed clinical symptoms of arsenic toxicity vary greatly, which poses a considerable challenge in relating the potential pathways of transfer of arsenic from groundwater to human metabolic system through food-chain. Although there may be several other factors involved in the relationship between ingestion of arsenic and epidemiology of arsenicosis, the significance of groundwater-arsenic ingested through the food route is not known. Along with intake of food, it is also possible that incidental ingestion and inhalation of dust containing arsenic may be a significant pathway of exposure (10).

This paper concentrates on arsenic contamination in food-chain through water-soil-crop route. The dietary habit of an individual, especially the nature and the amount of food eaten, might play some role in the arsenic dilemma. The extent of the role of arsenic in food-chain necessitates an in-depth study of bio-magnification of arsenic toxicity through the food-chain. The paper provides data on the contamination of soil through arsenic-contaminated irrigation-water and the subsequent transfer of arsenic via water/soil to crops. The findings are likely to help plan remedial measures to combat arsenic contamination in the food-chain through water-soil-crop transfer.

## MATERIALS AND METHODS

### Sampling sites

**Fig. 1.**
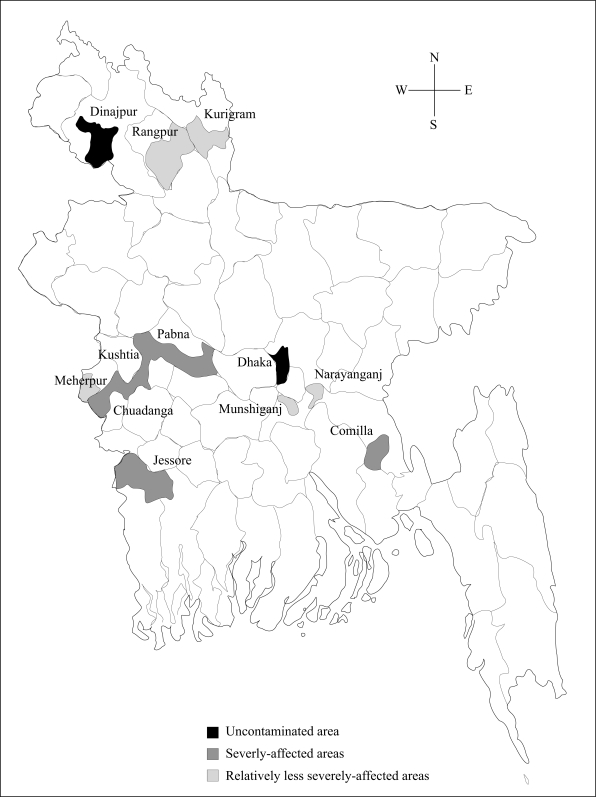
Sampling areas in different regions of Bangladesh

For the study, information about contamination of arsenic was obtained from secondary sources (1). Based on the information gathered, selected areas were identified as control (wherein arsenic contamination in groundwater was below the Bangladesh water-quality guideline of 50 μg/L), and as less-affected, moderately-affected, and severely-affected. Water, soil and vegetable/crop samples from 160 sites representing 15 districts were collected (fig. 1 and Table 1).

**Table 1.** Districts selected for collection of samples

Gangetic alluvium flood plain (severely-affected)	Teesta alluvium flood plain (less-affected)	Meghna Brahmaputra alluvium flood plain (severely to moderately-affected)	Pleistocene terrace (control)
Jessore	Rangpur	Comilla	Dhaka
Kushtia	Kurigram	Narayanganj	Dinajpur
Meherpur		Munsiganj	
Chuadanga		Noakhali	
Faridpur		Brahmanbaria	
Pabna			

### Water

Water samples (100 mL) from hand-tubewells or irrigation-pumps were collected from mid-stream by initially pumping water for five minutes. Immediately after sampling, one mL of concentrated HCl was added to the 100 mL vials containing water and transported to laboratory for further analysis. Vials were filled to the top and transferred to laboratory on ice within 24 hours. After this, the samples were centrifuged at 4,500 × g, filtered through a 0.45-μ millipore filter and analyzed for arsenic using hydride generation-atomic absorption spectrophotometry (HG-AAS).

### Soils

Replicate surface (0–15 cm) and sub-surface (15–30 cm) soil samples were collected from sites from where water and vegetables were collected and from where arsenic contamination in groundwater had been reported. Each replicate soil sample was a composite of 10 sub-samples (taken from the same depth). To monitor the load of arsenic on soils from water, samples were collected from regions having hand-tubewell, shallow tubewell, and surface-water irrigation. Grid sampling was adopted, and the number of samples collected from each site ranged from 25 to 40 per acre. After collection, samples were air-dried, ground, and screened to pass through a 0.5-mm sieve and stored in plastic vials for complete laboratory analysis.

### Vegetables

Replicate samples (usually 6 to 10) of edible parts of vegetables/crops commonly grown in the sampling area were collected. All plant samples were cleared of adhering soil particles, washed three times with de-ionized water and 0.05 M HCl, and then washed with de-ionized water three times to ensure dislodging and removal of dust particles. Samples were then dried in a fan-forced oven at 60±5 °C for 48 hours, ground using a stainless steel grinder, sifted through a 0.2-mm sieve, and stored in plastic vials for further analysis.

### Cooked rice

To assess the contribution of arsenic-contaminated water to cooked food, particularly to cooked rice (*bhat*), a laboratory experiment was carried out with 13 different rice samples procured from a local wholesale market. The sources of the rice samples were also noted. Arsenic contents in rice samples were determined following the procedure mentioned by Portman and Riley (12). All rice samples showed levels of arsenic below the detection level of the machine (2 μg/kg); 5.0 g from each rice sample was cooked in the laboratory following two conventional methods: Method I—50 mL of water and rice were cooked so that all the water was absorbed by the rice by the time it was well-cooked; Method II—100 mL of water was used, and when the rice was well-cooked, the liquid starch was decanted. The rice samples were cooked with fresh water or spiked water (equivalent to 50 μg/L of arsenic). All experiments were replicated thrice. The arsenic content of the cooked rice and the liquid starch were also estimated (12). The results were then extrapolated to the average consumption of cooked rice per person per day (450 g of uncooked rice).

### Laboratory analyses

Water-soluble and total arsenic content of soils was assessed following aqua-regia hot-plate digestion of sub-samples (0.5 g and 3 replicates) of soils. Soils were digested with three aliquots of aqua-regia (5 mL) solution. Following digestion, the extracts were diluted to 50 mL using aqua-regia, and arsenic in the extract was estimated by HG-AAS following calibration of the equipment. For every 10 soil samples, we included a certified reference material (CRM). Analyses of the extract showed that the digest reproduced 95% of the total arsenic content reported for the CRM (soil CRM was obtained from the Commonwealth Scientific and Industrial Research Organisation [CSIRO]), Adelaide, Australia; the quoted value was 1,200 mg/kg, and the recovered value was 1,150 mg/kg).

The total arsenic content of the plant material was estimated by HNO3 digestion (12). For plant materials, a CRM was included for every 10th sample to ensure quality assurance and quality control. (The CRM was also supplied by the CSIRO, and it was GBW 07603, average value 1.25 mg/kg with a range of 1.1–1.4 mg/kg; the recovered value ranged from 1.25 to 1.27 mg/kg). Ten sub-samples of soils and plant materials were also sent to another laboratory (CSIRO Land and Water, Adelaide, South Australia). There was good agreement (r=0.854, p=0.0) between the two laboratories.

In total, 2,500 samples of different vegetables, rice, wheat, and grasses from arsenic-affected and non-affected areas were collected and analyzed for determining the total levels of arsenic. All results expressed in the text are based on dry-weight basis.

## RESULTS

### Arsenic in soil

**Fig. 2.**
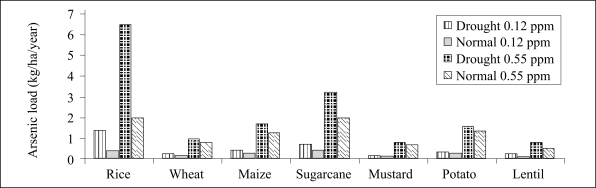
Arsenic load from irrigation-water for some cash crops

**Fig. 3.**
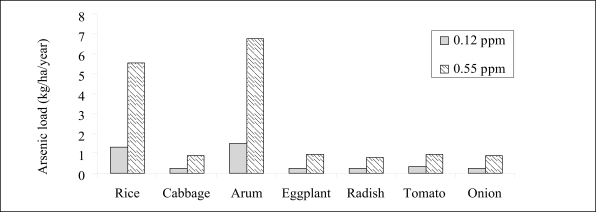
Arsenic load from irrigation water for some crops compared to that for rice

**Fig. 4.**
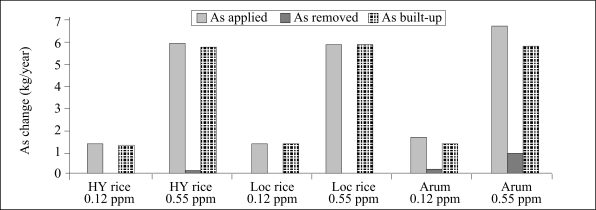
Dynamics of arsenic transfer through water-soil crop route

Concentration of arsenic in water used for irrigation varied from 0.136 to 0.55 mg/L. Using these values, total loading of arsenic in irrigated soils for a *boro* rice that requires 1,000 mm of irrigation-water per season ranged from 1.36 to 5.5 kg/ha/year. Similarly, for winter wheat that requires 150 mm of irrigation-water per season, loading of arsenic from irrigation ranged from 0.12 to 0.82 kg/ha/year. In addition, the loads of arsenic for other crops requiring irrigation were also calculated and are presented in Figures 2 and 3. The soil build-up of arsenic has been calculated based on loading of arsenic from irrigation, the average yield per hectare of some commonly-consumed crops, and the arsenic accumulation in the crops. Figure 4 shows that the build-up of arsenic in surface through irrigation-water, although not very high, is the greatest for arum (*Colocassia antiquorum*), followed by *boro* rice that requires supplemental irrigation.

Irrigation data obtained from Bangladesh Agricultural Research Council Arsenic in water from laboratory analysis

Irrigation data obtained from Bangladesh Agricultural Research Council Arsenic in water from laboratory analysis

Estimation is based on laboratory values, and data were obtained from Bangladesh Agricultural Research Council Irrigation water had 0.12 or 0.55 mg/L of arsenic Plant arsenic-content data were based on moderate and extreme cases Irrigation rates for rice and arum taken as 1 m and 1.2 m per crop respectively As=Arsenic; HY=High-yielding; Loc=Local

**Fig. 5.**
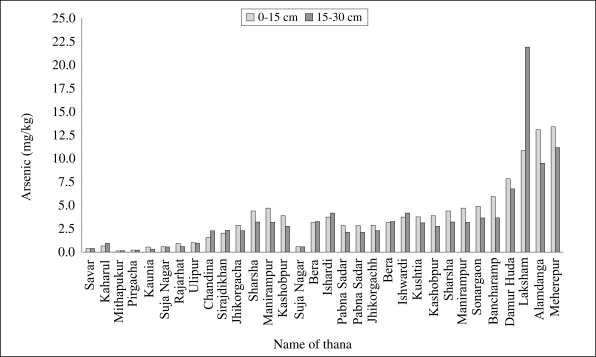
Arsenic contents in different depths of soils collected from arsenic-affected areas

Laboratory-based column studies done by the authors showed that 60–70% of arsenic applied in influent water containing arsenic similar in concentration to irrigation to the soils leached out of the column. However, the proportion of arsenic retained varied with soil texture and pH levels; soil with high pH showed low retention. In general, most soils contained <10 mg/kg of arsenic and, as such, met the guidelines for residential soils of 100 mg/kg as required by the Australian Health and 20 mg/kg as required by the environmental guidelines (13). Moreover, concentration of arsenic in surface (0–15 cm) soils typically exceeded sub-surface layer (Fig. 5). In some soils, aqua-regia extractable arsenic exceeded >50 mg/kg with the highest concentration being 81 mg/kg. The highest concentration was recorded for soil receiving irrigation from a shallow tubewell. At this site, the sub-surface soil contained about 3 mg/kg of arsenic, which indicates that arsenic added to the soil at this site through irrigation is concentrated in the top 0–15 cm layer. This layer corresponds to the main root-zone depth for most cultivated crops.

In soils contaminated through anthropogenic activity, arsenic contents may exceed 50 mg/kg. Ali *et al*. reported that arsenic accumulates in the soil of rice fields where higher levels are found in the top 75–150 mm layer (14). The concentration of arsenic in the irrigated soils varied from 3.2 to 27.5 mg/kg. On the other hand, in the areas where irrigation-water did not contain arsenic, the soil arsenic varied from 0.10 to 2.75 mg/kg. The results of this study showed that concentrations of arsenic in soil decreased with depth.

### Water-soil-crop route for transfer of arsenic

In general, arsenic content in plants varied considerably with type of plants, type of soil, and arsenic content of irrigation-water. Arsenic in similar type of plants was several times greater in arsenic-affected areas than in unaffected areas. The highest concentration of arsenic was recorded for the arum vegetable, and this ranged from <10 mg/kg to >100 mg/kg in the peeled root samples. The high content of arsenic in arum crops was similar to the data reported in both our earlier studies and also those by other researchers. The comparison of arsenic content in soil with arsenic content of arum did not reveal any significant relationship (p=0.234), indicating that levels of arsenic in soil do not dictate the arsenic-uptake capacity of arum plants; the very high concentration indicates the special capacity of this plant to bio-accumulate arsenic. However, when the analyses were conducted at district level, there was a non-significant positive correlation (r=0.162) between content of arsenic in plants and content of arsenic in soil.

Generally, the highest concentrations of arsenic were always recorded in plant-roots, and this may be attributed to contamination from fine colloidal particles. Peeled vegetable samples also showed concentration of arsenic higher than the Australian permissible levels (1 mg/kg fresh weight), indicating significant accumulation in the plant tissues.

**Fig. 6.**
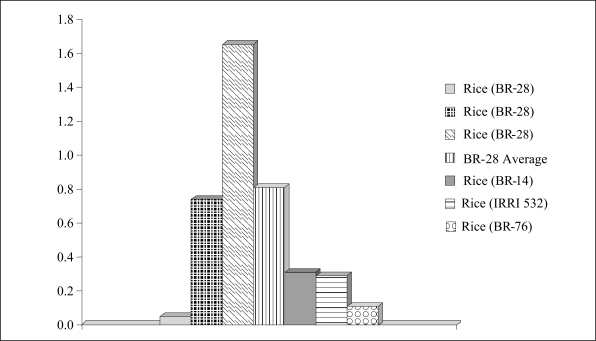
Arsenic content (mg/kg) in different rice varieties

Unlike arum, it was observed that the arsenic content in rice and wheat was mostly concentrated in the roots and straw. The arsenic content of rice grain samples collected from various districts varied from below detection limit to >1 mg/kg. The concentration in roots ranged from less than 1 to 267 mg/kg, while the range was from less than 1 to 30 mg/kg in straw. The values ranged from 0.5 to 1 mg/kg in wheat grain, from 0.2 to 30 mg/kg in straw, and from 1.5 to 3 mg/kg in root. Other investigators have also reported similar results (15–18). The arsenic content in rice grain varied according to the type and the area where it was grown (Fig. 6).

Of various crops/vegetables analyzed, not surprisingly green-leafy vegetables, including arum, gourd leaf, *Amaranthus* (*shak*, both data *shak* and *lal shak*), *Ipomea* (*kalmi*), act as arsenic accumulators with arsenic contents ranging from 8 mg/kg in gourd to 158 mg/kg dry weight in arum or 6 to 125 mg/kg fresh weight. Arum seems to be unique in that the concentration of arsenic can be high in every part of the plant. Arum, a green vegetable commonly grown and used almost everywhere in the country, is a very rich source of vitamin A and C and is usually grown in wet zones adjacent to tubewells. Analyses of arsenic in wet soils collected from areas adjacent to tubewells generally had higher phytoavailability of arsenic compared to soils from dry regions. Further detailed studies in our laboratory under glasshouse conditions revealed that uptake capability by arum differed depending on the type of arum.

**Fig. 7.**
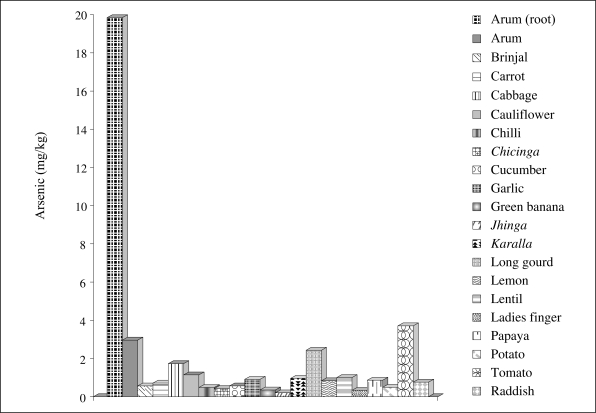
Arsenic content (mg/kg) in some commonly-grown crops collected from areas on Gangetic alluvium soil

**Fig. 8.**
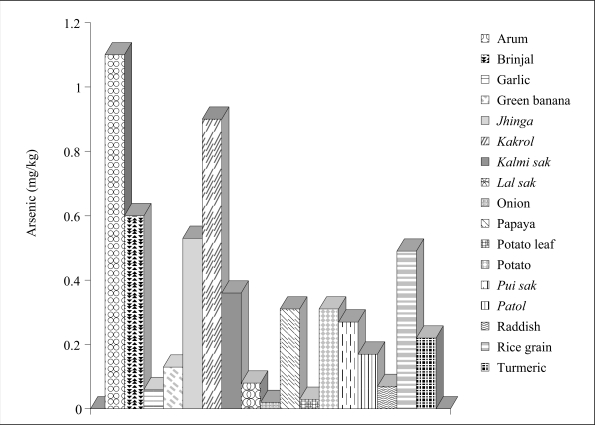
Arsenic content (mg/kg) in some commonly-grown crops collected from areas on Teesta alluvium soil

The average values for arsenic in different plant samples collected from the Ganga-Meghna-Brahmaputra (GMB) alluvium and Teesta alluvium are presented in Figure 7–8. A comparison of the two figures revealed that similar plants growing on contaminated soils of the Teesta alluvium had much lesser content of arsenic compared to those growing on the GMB alluvium. The marked difference in arsenic content of vegetables may be related to arsenic content of groundwater. The groundwater arsenic data revealed elevated arsenic in water draining the Gangetic or Meghna-Brahmaputra alluvium compared to the Teesta alluvium. This confirms the role of groundwater in arsenic content of crops.

There was a log-log relationship between concentration of arsenic in arum and concentration of arsenic in irrigation-water (r=0.78). On the other hand, the coefficient for soil arsenic was negative (Table 2).

**Table 2.** Regression of arsenic content of arum against soil arsenic and arsenic in irrigation water

Parameter	Coefficient	Standard error	*t*-statistic	p value
Intercept	1.4040	0.2959	4.7447	0.0002
Log water	0.7768	0.1628	4.7708	0.0002
Log surface soil	-0.5719	0.1784	-3.2059	0.0052

### Arsenic in fodder

Some grasses, used as fodder, were also found to have accumulated arsenic. Interestingly, in the samples collected from the Teesta alluvium, arsenic contents were relatively less than that in samples collected from the GMB alluvium. However, similar to the former case, samples collected from affected areas usually contained more arsenic than those collected from unaffected areas.

### Maximum allowable daily level in different crops

The maximum allowable daily level of arsenic in foodstuff is taken as 0.22 mg. Calculations were made based on the possibility of exceeding this maximum allowable daily level (MADL) for various plants analyzed (19). For example, a person who consumes 100 g of arum that contains 2.2 mg/kg of arsenic daily would have an MADL from arum alone. However, when the concentration of arsenic is as high as 22 mg/kg, only 10 g would give the MADL. Similarly, 440 g of rice with 0.5 mg/kg of arsenic would also represent the MADL. Such inputs are comparable to drinking 4.4 L of water with 0.05 mg/L of arsenic. The authors also analyzed some cooked foods for their arsenic content. These were collected from households who used arsenic-contaminated water for cooking. The foods contained appreciable amounts of arsenic in them (20).

The results relating to cooked rice indicated (Table 3) that, even if a rice sample did not contain any detectable amount of arsenic, the cooked rice (*bhat*) contained a substantial amount of the element arsenic when it was cooked with arsenic-contaminated water. The quantity was higher when Method I was followed to cook. Although Method II was a better method, there was still quite an elevated amount of arsenic. The differences in arsenic content in varieties of cooked rice by either of the methods could be attributed to the surface characteristics and the moisture absorbing/retention properties of rice varieties. Rice cooked with arsenic-free water did not show any detectable arsenic content. Cooked rice collected from households during the field survey showed concentrations of arsenic from 0.11 to 0.36 mg/kg (20). The amount of arsenic in cooked rice (*bhat*), plus an average consumption of four litres of drinking-water from the same source, with the Bangladesh standard of 50 μg/L of arsenic, is sufficient to bring the value of daily ingestion of arsenic above the MADL of 0.22 mg per day. Chakravarty *et al*. estimated that the content of arsenic ingested by a person from cooked rice (*bhat*) is 0.124 mg from 460 g of rice (23). It should also be noted that the bioavailability of arsenic in rice is quite high (9).

**Table 3.** Arsenic content in cooked rice by method of cooking

Rice variety	Source location	Arsenic in cooked rice *(bhat)* (mg/kg)		Arsenic in liquid starch (mg/kg)
		Method I	Method II	
Najirshail	Savar	0.048 (0.021)	0.023 (0.01)	0.068
Najirshail	Bogra	0.103 (0.046)	0.012 (0.006)	0.064
Najirshail	Dinajpur	0.060 (0.027)	0.030 (0.014)	0.094
Paripaijam	Nowga	0.137 (0.062)	0.040 (0.018)	0.059
Paripaijam	Natore	0.110 (0.05)	0.022 (0.01)	0.069
Paripaijam	Chapainowabgonj	0.088 (0.04)	0.021 (0.009)	0.056
Paripaijam	Dinajpur	0.267 (0.12)	0.092 (0.041)	0.080
BR28	Sherpur	0.077 (0.035)	0.036 (0.016)	0.095
Bashful	Shylhet	0.079 (0.035)	0.007 (0.003)	0.076
Minicate	Kushtia	0.108 (0.049)	0.017 (0.008)	0.062
BR28	Kushtia	0.076 (0.034)	0.033 (0.015)	0.064
Paijam	Sherpur	0.120 (0.054)	0.029 (0.013)	0.071
BR28	Mymensingh	0.068 (0.031)	0.035 (0.016)	0.071
SD at 5%		0.055 (0.025)	0.021 (0.009)	0.012

Figures in parentheses indicate arsenic (mg) in cooked rice equivalent to 450 g of uncooked rice

SD=Standard deviation

Sufficient information was available from dietary data to give an empirical distribution. Similarly, an empirical distribution for the concentration of arsenic in rice could be determined for rice from different districts. A convolution of the distributions was used for determining the proportion of people whose daily intake of arsenic from rice exceeds the MADL. The authors made a dietary survey to assess the exposure pathways of ingestion of arsenic. Based on data obtained for population of different districts, the percentage of the population at risk of exposure to excess MADL has been calculated for Jessore (representing Gangetic alluvium) and Rangpur (representing Teesta alluvium). In Jessore, 32% of the people were above the MADL, while the value was only 2% in Rangpur (19). This again substantiates the fact that groundwater in the Gangetic alluvium is more contaminated compared to other areas of the country. When extrapolated for the whole country (as represented by the average of the two areas), the value came to 19% of the population.

## DISCUSSION

In the present study, it has been observed that arum accumulates high amount of arsenic, particularly when growing around arsenic-contaminated tubewells. It is, thus, advisable that cultivation of arum is relocated to regions away from tubewells where wetting and drying cycles might have impact on phytoavailability of arsenic.

The present findings (Figs. 7 and 8) further revealed that, in many plants, arsenic content in their tissues are at elevated levels. It is also apparent that arsenic present in the growing environments of the plant is not completely phytotoxic. If the environments of the plants had been completely phytotoxic, those plants would not have survived. Similar findings of arsenic content of some common vegetables were recently reported by Farid *et al*. who found that the accumulation was greater in similar vegetables grown on soils belonging to the Gangetic alluvium compared to those growing on soils of the Teesta alluvium (21).

However, at this stage, there are few data that are adequate to assess whether sub-lethal concentrations of arsenic can cause small reductions in plant growth. These few and dietary data obtained in our study indicate that loads of arsenic from diet alone at times exceed the maximum daily intake of arsenic.

The toxic effect of arsenic in any foodstuff is highly dependent on its chemical speciation. Inorganic arsenic compounds are generally more toxic than organic forms. The toxicity of arsenic species follows the order AsH3 >As(III) >As(V) >MMAA (monomethylarsonic acid) >DMAA (dimethylarsinic acid). There are reports of transformation of arsenic species in the plant system (15). In a recent study, Williams *et al*. found that the mean arsenic level from Bangladeshi rice was 0.13 (range 0.03–0.30) mg/kg (24). The study also found that the main species detected in Bangladeshi rice were As(III), DMAV, and As(V), of which more than 80% of recovered arsenic was in the inorganic form. In a separate study, it was observed that 82% of rice arsenic was bioavailable (9). The Australian limit of 1 mg/kg dry weight was set with regard to the high seafood intake of Australian people (25), as seafood contains elevated amounts of arsenic. However, most arsenic in this seafood is organic (26). On the other hand, in Bangladeshi rice grain, arsenic is dominated by the more toxic inorganic form (24). The proportion of inorganic and organic arsenic in rice grain varies from sample to sample. A possible explanation for this is that arsenic is taken up by plant through irrigation-water or through a part of the soil where arsenic is soluble in water. Similar observations were made with pot-experiments (13). Much of arsenic in irrigation-water is in the As(III) form (20); thus, water entering the crops will also be in that form. The proportion of arsenic in the toxic As(III) form will depend on the rate of biomethylation in that species. Furthermore, just as the rate of biomethylation varies from individual to individual and also according to sex and age (22), there may be variation among biomethylation rates of different plant species.

To ensure complete safety, total arsenic must be assumed to represent the value of inorganic arsenic in rice until data on the speciation of arsenic in rice are robust enough to effectively predict the risk associated with consumption of rice (24). This statement will justify our attempt to relate total arsenic in food and its possible implications in the food-chain. Moreover, our assumptions regarding MADL with cooked rice and water are also supported by Williams *et al*. (24).

The above information asserts that ingestion of arsenic by humans can occur not only through drinking water but also through the food-chain. Crops receiving arsenic-contaminated irrigation-water take up this toxic element and accumulate it in different degrees depending on the species and variety. However, the portion of the arsenic that goes directly into different metabolic pathways and causes the problem of arsenicosis needs to be assessed. The bioavailability of arsenic in different food materials needs to be further assessed, and a screening of vegetables that contain exceptionally high amount of arsenic needs to be carried out. We need to ensure that we eat arsenic-free food. Much work still need to be done to gain a better picture of arsenic exposure among the people of Bangladesh. The preliminary results of our study suggest a further detailed study and development of strategies that minimize the water-soil-plant transfer of arsenic.

## ACKNOWLEDGEMENTS

This work was financially supported by the Australian Centre for International Agricultural Research, Australia and the Ministry of Education, Government of the People's Republic of Bangladesh.
